# High density of genuine growth twins in electrodeposited aluminum

**DOI:** 10.1126/sciadv.aax3894

**Published:** 2019-10-18

**Authors:** Lidija D. Rafailović, Christoph Gammer, Christian Ebner, Christian Rentenberger, Aleksandar Z. Jovanović, Igor A. Pašti, Natalia V. Skorodumova, H. Peter Karnthaler

**Affiliations:** 1 CEST Center of Electrochemical Surface Technology, Viktor-Kaplan Strasse 2, 2700 Wiener Neustadt, Austria.; 2 Erich Schmid Institute of Materials Science, Austrian Academy of Sciences, Jahnstrasse 12, 8700 Leoben, Austria.; 3 Physics of Nanostructured Materials, University of Vienna, Boltzmanngasse 5, 1090 Vienna, Austria.; 4 Faculty of Physical Chemistry, University of Belgrade, Studentski trg 12-16, 11158 Belgrade, Serbia.; 5 Department of Materials Science and Engineering, School of Industrial Engineering and Management, KTH–Royal Institute of Technology, Brinellvägen 23, 100 44 Stockholm, Sweden.; 6 Department of Physics and Astronomy, Uppsala University, Box 516, 751 20 Uppsala, Sweden.

## Abstract

We demonstrate electrodeposition as a synthesis method for fabrication of Al coatings, up to 10 μm thick, containing a high density of genuine growth twins. This has not been expected since the twin boundary energy of pure Al is very high. TEM methods were used to analyze deposited Al and its nanoscaled twins. DFT methods confirmed that the influence of the substrate is limited to the layers close to the interface. Our findings are different from those achieved by sputtering of Al coatings restricted to a thickness less than 100 nm with twins dominated by epitaxial effects. We propose that in the case of electrodeposition, a high density of twins arises because of fast nucleation and is additionally promoted by a monolayer of adsorbed hydrogen originating from water impurities. Therefore, electrodeposition is a viable approach for tailoring the structure and properties of thicker, deposited Al coatings reinforced by twins.

## Teaser

We demonstrate the synthesis of highly twinned Al using electrodeposition and propose a model for the continuous twin nucleation.

## INTRODUCTION

Nanostructured metals reveal a spectrum of extraordinary properties that are of current interest for a variety of structural and functional applications. In this context, the fabrication of nanostructured metals containing nanoscaled twins, both growth twins and deformation twins, is considered as a promising approach for the synthesis of high-performance materials. Several experimental studies and computer modeling results have demonstrated that nanoscaled metallic twining can lead to extraordinary physical properties (*1*, *2*). In face-centered cubic (fcc) metals, the formation of twins is strongly related to the stacking fault energy. In metals with a low stacking fault energy such as Ag and Cu, the formation of both deformation and growth twins is expected (*3*). In Cu, the formation of a high density of growth twins was demonstrated experimentally by electrodeposition and sputtering and has attracted wide interest due to the attractive properties of the resulting material (*4*, *5*). Besides the work of Lu *et al.* showing formation of nanotwins in fcc metals with a low stacking fault energy (γ_sf_) as Cu, the formation of twins as a kind of two-dimensional defect has been observed and reported for electrodeposited dense Ni layers and their alloys (*6*–*9*). Early transmission electron microscopy (TEM) investigations determining the energy of the stacking fault of fcc materials showed that small alloying additions can change the stacking fault energy considerably, e.g., adding 5 atomic % Al to Cu reduces the stacking fault energy of pure Cu (value of γ_sf_ = 41 ± 9 mJ m^−2^) (*10*) by about 50%, and similarly, the addition of nonmetallic Si leads to a reduction of γ_sf_ (*11*, *12*). In addition, twinning was observed in Ag electrodeposits especially when produced under conditions far from equilibrium (*13*). It is interesting to note that in contrast to electrodeposited layers, those achieved by magnetron sputter deposition show much less grown in twins (*3*). Nanoscale growth twins were mainly achieved by sputtering in the case of Cu and stainless steel (*3*, *14*); in contrast, growth twins were reported in Ni only by alloying (*15*).

Because of the very high stacking fault energy of Al, γ_sf_ = 120 to 165 mJ m^−2^, twins grown in Al were, until recently, considered as highly unexpected or even not possible to achieve (*3*, *16*). The twinnability in Al is very low compared with other fcc metals and, generally, is not favored as a deformation mechanism in high-symmetry cubic materials (*17*). In the case of nanocrystalline metals, twinning was predicted and even observed in metals with high γ_sf_, such as Al, under extreme conditions like deformation (*2*, *18*), and in low density as recrystallization twins observed at high temperatures or by using a low stacking fault interface layer (*16*, *19*). The formation of a high density of growth twins, however, seems unlikely but was recently accomplished by magnetron sputtering by Bufford *et al.* (*19*) in the special case of Ag/Al multilayer films. An Ag seed layer that readily forms twins, owing to a low stacking fault energy of 16 mJ m^−2^, was repeatedly sputtered to induce twins in the subsequently sputtered Al layers by epitaxy (*16*, *19*). This approach enables the formation of complex epitaxial multilayered structures composed of Ag and Al layers with thicknesses of the alternating layers from 100-nm Ag/100-nm Al to 5-nm Ag/100-nm Al (*16*, *19*). Electrodeposition of Al, on the other hand, has not achieved sufficient attention and, to our knowledge, was not used to date as a route toward nanotwinned Al (*20*, *21*).

In the present work, we demonstrate the use of electrodeposition as a synthesis method to make Al layers with a high density of genuine growth twins. This unexpected result demonstrates a versatile and cost-effective nanoengineering technique for the formation of pure Al layers with a high density of twins, showing attractive properties that could be of particular interest in aerospace for tribological applications as a possible replacement for toxic Cd (*22*). Replacing grain boundaries by twin boundaries increases the conductivity, and this might be advantageous in, for example, the case of Li-ion battery applications with Al used as a current collector. Last, we suggest that the presented electrodeposition process could be generalized as a strategy for obtaining twinned deposits of high stacking fault energy metals with larger thickness.

## RESULTS

Cyclic voltammetry experiments (fig. S1) show an irreversible behavior for Al deposition on the prepared Ag substrate with an overvoltage of −120 mV versus Al needed to start the nucleation of the Al deposit. Considering that Al is deposited from an electrolyte that contains a very high concentration of AlCl_3_ (60 mole percent), diffusion is not solely rate determining in this case. According to the chronopotentiometric curves for two electrodeposition currents (cf. fig. S2), it can be seen that the electrode depolarizes quickly in the initial stages of electrodeposition, but the potential reaches nearly stationary values with small oscillations during the deposition.

**Fig. 1:**
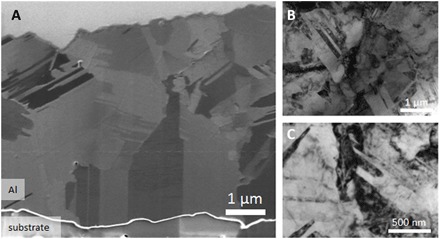
Electron microscopy investigations of the electrodeposited Al layer. (**A**) SEM image of a cross section through the Al deposit, revealing micrometer-sized grains containing a high density of twins. The bright line at the bottom indicates the interface between the deposited Al film and the substrate (Ag paste). (**B**) TEM image taken from a top-view specimen, revealing a high density of twins. (**C**) This is also confirmed in the TEM image taken from a cross-sectional specimen.

The morphology and microstructure of the obtained Al layer electrodeposited at −20 mA cm^−2^ were studied by advanced electron microscopy methods. Figure 1A shows a scanning electron microscopy (SEM) image of a cross section of the Al deposit on the Ag substrate, achieved using focused ion beam (FIB) cutting. The well-annealed Ag substrate (fired at 850°C, close to the melting temperature, *T*_m_ = 962°C, for 10 min) shows a large grain size and only few wide recrystallization twins (more than 1 μm). It is interesting to note that the coherent twin boundaries extend from the Ag substrate to the adjacent grain of the Al layer; the small shift observed is due to the fact that there is a height step at a boundary (cf. Materials and Methods). Further away from the substrate, epitaxy breaks down, and multiple new randomly shaped grains form. These grains contain a high density of twins, indicated by contrast features with sharp lines in the SEM image. The formation of extended twinning during further growth is quite unexpected. To analyze the microstructure with higher resolution, we used TEM methods. TEM bright-field images of the Al layer from both the top view and cross section are given in Fig. 1 (B and C, respectively). The top-view sample shows grains with a size of around 1 μm containing a high-density of dislocations and twins (cf. Fig. 1B); the cross-sectional TEM image reveals a similar grain size and twin spacing (cf. Fig. 1C). The grains are rather equiaxed. The twin spacing was measured from both TEM and SEM images using the line spacing method, revealing a broad distribution from 20 to 500 nm with a mean value of around 100 nm. For comparison, we also present an SEM image of the Al deposit produced at a lower current density (−10 mA cm^−2^; fig. S3); which shows qualitatively a similar trend to the deposit formed at −20 mA cm^−2^ but with a lower concentration of twins.

**Fig. 2:**
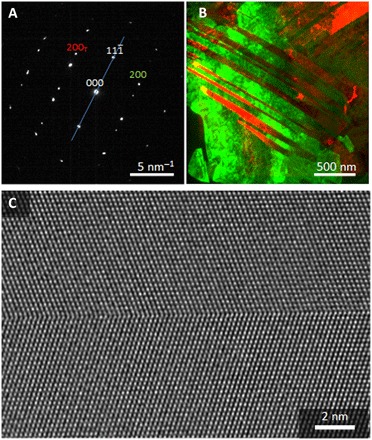
TEM investigation of the electrodeposited Al specimen (top view). (**A**) Diffraction pattern ([110] orientation) taken from a twinned grain. The mirror plane (blue line) and a twin reflection (subscript T) are marked. (**B**) Color-coded overlay of dark-field images generated using the reflections marked green (200) and red (200_T_) in (A). (**C**) High-resolution TEM image showing the atomic structure of a twin boundary.

Figure 2 shows a detailed TEM investigation of an electrodeposited grain (*j* = −20 mA cm^−2^) away from the interface with the substrate. The diffraction pattern of the grain reveals that it has a [110] zone axis orientation and contains a (111) twin boundary (cf. Fig. 2A). The twin reflection is indicated by using the subscript T. Figure 2B shows a color-coded overprint of two TEM dark-field images taken with matrix and twin reflections (as indicated in Fig. 2A), demonstrating that the grain consists of alternating twinned lamellae. To investigate the atomic structure of the twins, we took high-resolution TEM (HRTEM) images of (111) twin boundaries recorded in [110] orientations, revealing that the twin boundary is atomically sharp (cf. Fig. 2C).

To determine the purity of the Al deposit, we carried out a careful chemical analysis. Special attention was given to metallic impurities since they influence the stacking fault energy and therefore might affect the formation of the twin boundaries. We recorded and analyzed the composition of the electrodeposited Al layer with energy-dispersive x-ray spectroscopy (EDS) in the TEM by two-dimensional maps containing a full EDS spectrum for each pixel.

Table S1 shows the average composition determined from the summed EDS spectrum. The trace elements measured in the specimen include several elements specifically due to specimen preparation and also those caused by the TEM instrument. This includes Mo from the TEM grid, Cu from the TEM sample holder, Ar from ion milling, and, additionally, Fe from the TEM pol pieces. The oxygen detected is considered to arise from surface oxidation of Al since the specimens show no indication that they contain crystalline alumina. Only the measured chlorine is attributed to the electrolytic deposition process as the electrolyte used for the synthesis contains Cl. Last, no metallic impurities and no enrichment of impurities were detected at the twin boundaries, as demonstrated by the composition profiles measured by EDS (cf. table S1). These results demonstrate both the high purity of our deposit and rule out a possible change of the stacking fault energy as caused by impurities.

**Fig. 3:**
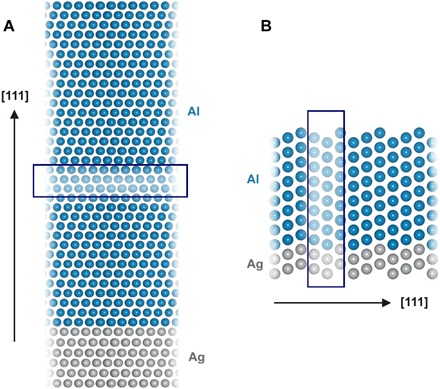
Construction of supercells used for simulations. (**A**) Hexagonal supercell (1 × 1), constructed by stacking 40 (111) planes. In this image, the supercell is repeated normal to the [111] direction to better show the marked defect structure. (**B**) Simulation cell used in the twin propagation calculations, with the twin position Ag (bottom; gray) and Al (top; blue) marked. Images were generated using VESTA (*53*).

To obtain an insight into the role of the Ag substrate (cf. fig. S4) on the twinning process in the electrodeposited Al, we carried out density functional theory (DFT) calculations. First, we investigate how the nucleation of a twin away from the interface with the substrate could be influenced by the substrate. Therefore, we used DFT calculations to analyze how the Ag substrate affects the stacking of Al layers as the function of the distance from the interface with the Al substrate. For this purpose, we used the supercell constructed as shown in Fig. 3A. In these calculations, we assume that the Al overlayer is adapted to the Ag lattice parameter. This is justified since the lattice parameter of Al (calculated to be 0.404 nm) differs from that of Ag (0.416 nm), but not much. Hence, the Al deposit is subjected to a small strain of 2.5%, not affecting the conclusions drawn from the calculations. By comparing the results of the calculations with the Ag substrate with those without the Ag substrate, we could not observe any specific impact of the Ag substrate on the Al stacking fault energy more than three layers away from the Ag/Al interface region. This is in agreement with the recent findings that the effect of substrate for fcc overlayers is lost already after the third layer (*23*)). From these results, we can conclude that the twinning in the grains far off the deposit is due to the electrodeposition process and not linked to the interface. We also used our present DFT calculations to estimate the stacking fault energy of pure Al and got the result, γ_sf_ = 170 mJ m^−2^, which is in good agreement with the values published in the literature (*24*–*26*).

Second, we also used DFT calculations to investigate how twins in Ag can propagate into the Al deposit. For this purpose, we constructed a cell, as shown in Fig. 3B. In these calculations, we adapted the lattice parameter of the Al overlayer again to that of Ag. For different thicknesses of the Al deposits, we constructed cells with different combinations of coherent twin boundaries in the Ag substrate and Al deposit (cf. fig. S5). When the systems are fully relaxed, the comparison of the total energies reveals that a configuration with a twin in Ag that continues into the Al deposit (cf. fig. S5) is favored as compared with the case with pristine Al growing on an Ag substrate with a twin. This shows that the propagation of coherent twins from Ag to Al is energetically favorable. In addition, a partial reconstruction of an assumed Ag(twin)/Al(pristine) interface to an Ag(twin)/Al(twin) configuration was observed during the structural relaxation. These results indicate that the properties of the substrate are decisive in the initial deposition steps. Therefore, DFT calculations agree with our experimental finding that the few twins found in the annealed Ag substrate propagate into the epitaxial Al grains formed directly at the interface.

**Fig. 4:**
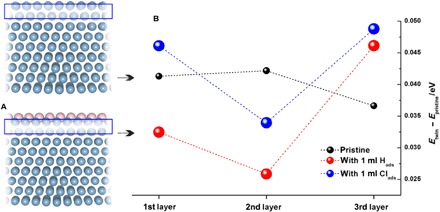
Effect of (surface) adsorbates, H_ads_ and Cl_ads_, on Al twinning, according to DFT calculations. (**A**) Surface models used to study the effects of adsorption. (**B**) The difference between total energies of twinned and pristine Al(111) in the case of no adsorbate on the surface and when the surface is saturated with 1 ml of H_ads_ or Cl_ads_. The position of the stacking fault is varied with respect to the surface layer.

Besides addressing the processes at the Ag/Al interface, we used DFT calculations to investigate possible processes at the Al/electrolyte interface. Since a very small amount of hydrogen cannot be avoided during electrodeposition even in the case of Al obtained from basically “water-free” electrolytes, i.e., ionic liquids, we carried out calculations assuming the existence of a monolayer of hydrogen adsorbed on the surface. The model for the atomic structure used for the calculation is shown in Fig. 4A. The resulting influence of the adsorbed layer of hydrogen on the stacking fault energy of Al is given in Fig. 4B. On the basis of the results of the DFT calculations, hydrogen leads to a reduction of the fault energy that is restricted to the first and second layers beneath the surface. Although the negative potentials at which deposition is performed reduce the possibility of chloride adsorption (originating from parasitic or anodic reactions of imidazolium-based chloroaluminates) (*27*), we note that Cl was observed in the sample by chemical analysis (table S1). Hence, in a separate set of calculations, we considered the effect of the Cl monolayer on Al twinning (Fig. 4B). In this case, the influence on the fault energy is but little and opposite to that of hydrogen when the stacking fault is at the first layer.

To evaluate the hardness of the electrodeposited layer, we carried out nanoindentation on the cross section of the twinned Al layer. Figure S6 shows the hardness of the electrodeposited layer as a function of depth. The achieved hardness is more than 900 MPa and therefore even higher than that found in the literature for commercially pure Al (about 600 MPa) processed by high-pressure torsion deformation (*28*). This result is a direct consequence of a high density of twins in electrodeposited Al.

## DISCUSSION

Nanotwinning is a promising approach toward high-performance materials, but in the case of high stacking fault energy metals, growth twins are still a rather rare and unexplored phenomenon. Until now, the high stacking fault energy of Al was a barrier to achieve a high density of twins in epitaxial multilayer specimens with Al layers thicker than 100 nm (*16*, *19*). A strategy to use Ag as a substrate template material to induce twins by epitaxial growth into Al is based on the similarity of their crystal structures and lattice parameters, although their stacking fault energies are significantly different. This approach was applied by sputtering synthesis using a seed layer of {111} Ag containing a high density of twins. Recently, studies on the formation of growth twins in Al by sputtering without the use of a twinned seed layer were presented but were again limited to thin layers as the increase of the coating thickness to more than 80 nm results in a rapid decrease in twin density. As a possible reason, the authors proposed a transformation of growth from island coalescence to continuous growth (*29*, *30*).

In the present work, electrodeposition is used as an effective alternative synthesis approach toward nanotwinned Al. In contrast to sputter deposition, newly nucleated grains far from the interface show a high twin density. In low stacking fault energy Cu, direct current and pulse deposition regimes were used to form a high density of twins, mainly coherent twin boundaries (*4*). Similar to sputtering, a deposition rate far from thermodynamic equilibrium, as reflected by its overpotential, can yield twinned deposits. Therefore, it was concluded that twins can form during electrodeposition at small current densities only when the overpotential is higher than some critical value. As indicated in the literature, a correlation exists between the overpotential necessary for the formation of twins and the twinning energy: the lower the twin boundary energy (e.g., in Ag), the lower the required overpotential (*31*). In this case, the formation of twins (having a small twin boundary energy) is preferred to that of high-angle grain boundaries. Our DFT calculations yielding a high value (80 mJ m^−2^) of the twin boundary energy for the case of Al indicate that the overpotential to form twins should be higher than that of Ag. This corresponds with the overpotential of −120 mV versus Al reference electrode to nucleate twins according to our cyclic voltammetry curve (cf. fig. S4).

It should be pointed out that Al dendrites are not formed, indicating that deposition is still below the limiting current density region. The surface of the obtained Al is somewhat rough but compact and dense as visible from the FIB SEM cross-sectional results (cf. Fig. 1A). These results can be compared with the case of Al deposition on a glassy carbon substrate (*32*), where coarse-grained deposits were obtained at current densities similar to the ones used here but at much more negative potentials (below −2 V versus Al). We ascribe these differences to the weak adhesion of Al to the carbon surface. That is, if graphene is taken as a model of the carbon surface, Al binds to it with an adsorption energy of only −0.88 eV (*33*). In comparison, we calculated the Al adsorption energy on Ag(111) to be −2.54 eV. Hence, we conclude that on a glassy carbon, Al would prefer island growth with large overpotentials needed for nucleation, while on Ag substrate, compact layer forms initially at moderate overpotentials (as compared with the case of a glassy carbon substrate) due to suitable Ag-Al interactions.

Our DFT calculations show that twin continuation in Al only occurs when a twin is present in Ag and the influence of the substrate is only notable in the first three overlayers. Nevertheless, we show in the experimental study that pronounced twinning is accomplished during further continuous growth at a constant deposition rate in electrodeposited Al at a distance far (≥1 up to 10 μm) from the Ag interface. Extensive twinning in a 10-μm-thick deposit is visible in the cross-sectional SEM image in Fig. 1A. This means that a high density of nanotwins forms in the grains nucleated in the Al deposit far from the substrate; only at the interface with the substrate a low density of twins grows epitaxially triggered by the few broad recrystallization twins present in the Ag substrate, which was fired at 850°C (cf. Fig. 1A). In addition, it should be pointed out that in recrystallized Al, such as in other fcc metals, recrystallization twins occur at a low density only, thus excluding the possibility that the observed high density of nanotwins is recrystallization twins. In addition, in the present case, there is no indication that the twins could be facilitated by metallic contaminations, reducing the stacking fault energy of the deposited Al. Hence, the electrodeposition process itself seems to be the main factor influencing the twinning of the formed Al deposit. We note that twinning is assumed to be more probable when the nucleation probability is higher (*31*). Under the conditions of electrodeposition, this translates to a higher (i.e., more negative) overpotential. Hence, deposition under lower overpotential (i.e., closer to thermodynamic equilibrium) would lead to a lower concentration of twins. This is confirmed by inspecting the deposit formed at −10 mA cm^−2^ (cf. fig. S3), which showed less pronounced twinning than the sample formed at −20 mA cm^−2^.

Both experimental results and DFT calculations show that the structure of the Ag/Al interface is not responsible for the existence of twins at large distances from the substrate. On the other hand, the investigation of interfacial processes at the Al/electrolyte interface is much more complicated using existing experimental methods. For this purpose, we used DFT calculations to investigate the effect of surface H and Cl adsorbates on the twinning process (Fig. 4). On the basis of the obtained results, we propose that the presence of an adsorbed layer of hydrogen during electrodeposition facilitates the nucleation of twins even far away from the substrate. It seems safe to assume that the further growth of the twins occurs by the replication of the faulted structure (cf. Fig. 3). The importance of the continuous nucleation is also reflected by the fact that, in our case, the density of twins increases as a function of the distance from the interface and does not correlate to the density of twins in the substrate any longer. It should be noted that the less pronounced twinning at lower current density (cf. fig. S3) indirectly supports the proposed mechanism of combined contribution of nucleation rate and hydrogen-promoted twin formation. That is, at more positive potentials (fig. S2), both nucleation rate and hydrogen surface concentration are lower, resulting in a lower concentration of twins.

At this point, a comparison with electrodeposited Cu and Ni can be made (*3*, *4*). In the case of direct Cu deposition at low current densities, one would not expect evolution of hydrogen. The addition of acid to the electrolyte leads to the formation of hydrogen, and it was reported that with increasing hydrogen content, the density of twins increases (*34*). This seems to be similar to the case of pulse Cu deposits, where twins and the presence of hydrogen were observed (*35*). In the case of electrodeposited Ni, the influence of hydrogen is expected since Ni is a catalyst for hydrogen evolution, which could be correlated to twin-related grains grown epitaxially on Cu substrate (*36*). In our case, direct evaluation of this hydrogen effect is difficult as introduction of protons as a source for hydrogen would inevitably result in further water contamination. This, in turn, leads to the formation of an Al(OH)_3_ passivating layer, which further suppresses Al growth (*37*). As a note, in our experiments, water content was around 10 parts per million (ppm) (see Materials and Methods), while the mentioned passivation effect occurs when a nondried electrolyte is used (300 ppm of water).

During sputter deposition, the twinning probability is mainly driven by the stacking fault energy. Therefore, because of the high stacking fault energy of the Al twin, nucleation is unlikely to occur, in agreement with literature reports. In the case of thin (≤1 μm) sandwiched layers by Bufford *et al.*, the high density of twins present in Ag is replicated almost exactly at the boundary with the Al layer. Consequently, the formation of twins in Al requires the deposition of Al onto a seed layer of Ag already containing a high density of twins (*16*, *19*). In the case of Al films on other substrates, the twin density shows a continuous decrease with the increase in the film thickness and an exhaustion at about 80 nm (*29*, *30*). The decrease in twin density with thickness is in contrast to the case of the electrodeposition process, where the interfacial processes can promote twin nucleation and thus result in an increasing twin density with increasing layer thickness (Fig. 1A). As revealed by cross-sectional SEM images and the DFT studies, the substrate can only influence the growth of deposited Al in the vicinity of the interface. In the later stages of electrodeposition, Al itself is acting as a substrate for subsequent growth, and twin nucleation occurs continuously at the Al/electrolyte interface, as suggested by DFT calculations (Fig. 4). Therefore, on the basis of the presented model, it is proposed that a high density of twins observed in the case of electrodeposited Al is a genuine effect of the electrodeposition process, where the presence of hydrogen can additionally promote twin nucleation. The importance of hydrogen on the stacking fault energy, twinning, and phase transformation was already observed in the case of the stainless steel (*38*–*41*); an increase in intensity of deformation twinning in fcc stainless steel was observed in samples charged with hydrogen (*42*). Therefore, we suggest that also in the case of electrodeposits, the importance of hydrogen on twinning should not be neglected.

In conclusion, it seems that conditions far from thermodynamic equilibrium solely are not sufficient for the twin formation in Al, as sputter deposition is carried out also at nonequilibrium conditions. Similarly, conditions away from thermodynamic equilibrium can also be achieved by severe plastic deformation as high-pressure torsion (*28*). Although Al processed that way has an even smaller grain size as compared with that of electrodeposited Al described here, it does not contain deformation twins. Nevertheless, in the case of electrodeposition, one must not disregard the existence of the Al/electrolyte interface and the associated interfacial processes. On the basis of the obtained results, we propose that the condition far from thermodynamic equilibrium during the electrodeposition allows fast nucleation, which is beneficial for twin formation, while the formation of the surface hydrogen phase augments the twin growth, which occurs by the replication of the faulted structure. From the practical point of view, because of the enhanced hardness of the deposit, electrodeposition of Al seems as a viable approach for the production of high-purity Al reinforced by twins.

## MATERIALS AND METHODS

### Preparation of Ag substrate and electrochemical synthesis of twinned Al

The Ag electrode used as a working electrode was made of a special mixed bonded silver paste (9912-A) applied on ceramic alumina with a thickness of 25 μm. An SEM image showing the microstructure of screen-printed Ag on ceramic electrode is provided in fig. S4B. Al was electrodeposited galvanostatically at a current density of 20 mA cm^−2^ for 30 min from an ionic liquid mixture of 60 mole percent of AlCl_3_ and 40% 1-ethyl 3-methylimidazolium chloride ([EMIm]Cl; BASF) (*43*), ensuring Lewis acidity. An ionic liquid was dried under vacuum with intensive stirring for 12 to 24 hours at temperatures that were kept in the range of 85° to 95°C to remove residual moisture. The content of moisture in the ionic liquid was checked by Karl Fischer coulometric titration showing water content before and after drying under vacuum of 300 and 10 ppm, respectively. After drying, the ionic liquid was transferred to an argon-filled glove box (LABstar MBraun) with O_2_ and H_2_O contents below 1 ppm where electrodeposition experiments were carried out. It should be emphasized that the ionic liquid used for electrodeposition was highly hygroscopic, and therefore, some residual water content was inevitable. The current densities of −10 and −20 mAcm^−2^ were selected because at lower values, longer deposition times are needed, which increased the risk of contamination, whereas at the higher current densities, the achieved layers were mechanically unstable. The preparation of the bath, the cyclic voltammetry measurements, the chronopotentiometric measurements, and the electrodeposition experiments were carried out inside of a glove box filled with argon at a temperature of 85°C using a VoltaLab potentiostat, PGZ 301. A pure Al sheet (99.999%, Goodfellow) was immersed into the ionic liquid and used as anode in constant current deposition experiments. The distance between the cathode and anode was about 2 cm. The electrolyte was stirred at a constant rate of 300 rpm. The cleaning procedure of the samples after electrodeposition and removal from the glove box includes washing with acetone, rinsing with deionized water, and drying with compressed air. The thickness of the obtained Al layer was around 10 μm.

### Electron microscopy measurements

To analyze the structure and composition of the sample, electron microscopy was used. Scanning electron microscopes FEI Quanta 200, equipped with an FIB, and Zeiss Supra 55 VP were used for top-view and cross-sectional imaging of the specimen. Since FIB ablation rates of Ag and Al were different, a height step at a twin boundary was formed, which led to a shift in the image as observed in Fig. 1. In addition, detailed TEM was carried out. To prepare plan-view TEM specimens, the Al film was peeled from the substrate and thinned to electron transparency using ion milling. In addition, a cross-sectional TEM lamella was extracted from the specimen by FIB methods. TEM images and diffraction patterns were acquired to analyze the grain and twin structure. In addition, aberration-corrected HRTEM, JEOL 2100F, was used to obtain atomic resolution images. Last, composition maps of the specimen were recorded in a scanning TEM, FEI Titan, using the superX four detector EDS spectrometer enabling to obtain quantitative maps with nanometer resolution and high-count rates. Quantification was carried out using the Bruker Esprit software.

### Mechanical measurements

Nanoindentation was performed on the cross section of the electrodeposited Al film as well as on a cross section of a commercial pure Al foil. Both cross sections were embedded into resin and carefully polished to obtain a smooth surface suitable for the indentations using 0.3-μm diamond polishing cloths. The nanoindentation was carried out using an ASMEC Unat with a Berkovich tip. To test for surface effects, depth dependence profiles of hardness and Young’s modulus were acquired using the quasi-continuous stiffness measurement method. Indents were carefully positioned under an optical microscope on the surface of the thin film. At least 25 points were taken for all measurements. A maximum load of 10 mN was used, and a hold segment was introduced at maximal load for 30 s to check for creep as well as a 60-s hold segment at 10% of maximal load to correct for thermal drift. The evaluation of the indentation data was performed according to the Oliver-Pharr method (*44*) using a Poisson’s ratio of ν = 0.33.

### DFT calculations

The DFT calculations were performed using the PWscf code of Quantum ESPRESSO (*45*), within the generalized gradient approximation of Perdew, Burk and Ernzerhof and ultrasoft pseudopotentials (*46*). The plane-wave kinetic energy cutoff was 36 rydberg, while the charge density cutoff was 16 times higher. First, we studied pristine bulk Ag and Al, as well as Ag and Al containing intrinsic stacking faults. The simulation cell corresponded to the unit cell for pristine bulk calculations, and a Γ-centered 12 × 12 × 12 Monkhorst-Pack *k*-point grid was used (*47*). Twin formation in both pristine metals was investigated in a hexagonal 1 × 1 supercell, constructed by stacking 39 (111) planes along the *z* axis of the cell. Next, the influence of the Ag substrate on the twin formation in Al was explored using a hexagonal 1 × 1 supercell, constructed by stacking 40 atomic layers along the [111] direction (Fig. 3A). Supercell dimensions corresponded to optimized bulk Ag lattice constants. The first six (111) planes were composed of Ag atoms, fixed during the structural relaxation steps, representing the substrate. The remaining layers consisted of Al (111) planes, containing a single stacking fault at varying distances from the substrate. In the final step, we examined the twin propagation from the substrate to Al during the initial electrodeposition steps. A supercell with six atomic layers of Ag, normal to the [111] direction, was constructed, upon which Al layers of varying thicknesses were added (Fig. 3B). The first irreducible Brillouin zone for calculations performed in hexagonal cells was sampled on a 7 × 7 × 1 (Fig. 3A), i.e., 7 × 1 × 7 (Fig. 3B) Monkhorst-Pack *k*-point grid. Periodic slabs in the last two calculations were separated by a vacuum layer, and to minimize the interaction between periodic images, a dipole correction to the electric field was applied, as described in (*48*). To investigate the effect of adsorbates on the twinning process, the surface was saturated with a full monolayer of H_ads_ or Cl_ads_ (assumed to originate from water impurities or the ionic liquid used in the experiment). The position of the stacking fault was varied with respect to the surface layer, and then the total energies of the pristine structure and the one with the stacking fault were compared.

## Supplementary Material

http://advances.sciencemag.org/cgi/content/full/5/10/eaax3894/DC1

Download PDF

High density of genuine growth twins in electrodeposited aluminum

## Supplementary Materials

Supplementary material for this article is available at http://advances.sciencemag.org/cgi/content/full/5/10/eaax3894/DC1 Fig. S1. Cyclic voltammogram recorded on Ag substrate. Fig. S2. Potential time curves obtained at different current densities on Ag substrates in AlCl_3_-[EMIm]Cl ionic liquid at 85°C. Fig. S3. SEM image of Al deposit formed at a current density of −10 mA cm^−2^. Fig. S4. SEM images of Al interface and screen-printed Ag substrate. Fig. S5. Supercell used in bulk defect calculations and different combinations of twinning. Fig. S6. Nanoindentation on cross sections obtained on electrodeposited Al sample and a commercial high-purity Al foil. Table S1. Average composition measured by EDS from the top-view TEM sample. Table S2. Calculated lattice constants, cohesive energies, twin formation energy, and intrinsic stacking fault energy. References (*49*–*52*)

## References

[1] Wang J., Zhang X., Twinning effects on strength and plasticity of metallic materials. MRS Bull. 41, 274–281 (2016).

[2] Beyerlein I. J., Zhang X., Misra A., Growth twins and deformation twins in metals. Annu. Rev. Mat. Res. 44, 329–363 (2014).

[3] Bufford D. C., Wang Y. M., Liu Y., Lu L., Synthesis and microstructure of electrodeposited and sputtered nanotwinned face-centered-cubic metals. MRS Bull. 41, 286–291 (2016).

[4] Lu L., Shen Y., Chen X., Qian L., Lu K., Ultrahigh strength and high electrical conductivity in copper. Science 304, 422–426 (2004). 15031435 10.1126/science.1092905

[5] Jiang H., Klemmer T. J., Barnard J. A., Doyle W. D., Payzant E. A., Epitaxial growth of Cu(111) films on Si(110) by magnetron sputtering: Orientation and twin growth. Thin Solid Films 315, 13–16 (1998).

[6] Noda H., Shibata A., Sone M., Ishiyama C., Higo Y., Characterization of texture and microstructure of electrodeposited Ni layers. MRS Proc. 1112, E05-01 (2008).

[7] Meng G., Shao Y., Zhang T., Zhang Y., Wang F., Synthesis and corrosion property of pure Ni with a high density of nanoscale twins. Electrochim. Acta 53, 5923–5926 (2008).

[8] B. Y. C. Wu, P. J. Ferreira, C. A. Schuh, in *Metallurgical and Materials Transactions A: Physical Metallurgy and Materials Science* (2005), vol. 36, pp. 1927–1936.

[9] Góral A., Nanoscale structural defects in electrodeposited Ni/Al_2_O_3_ composite coatings. Surf. Coatings Technol. 319, 23–32 (2017).

[10] Cockayne D. J. H., Jenkins M. L., Ray I. L. F., The measurement of stacking-fault energies of pure face-centred cubic metals. Philos. Mag. 24, 1383–1392 (1971).

[11] Gallagher P. C. J., The influence of alloying, temperature, and related effects on the stacking fault energy. Metall. Trans. 1, 2429–2461 (1970).

[12] Carter C. B., Ray I. L. F., On the stacking-fault energies of copper alloys. Philos. Mag. 35, 189–200 (1977).

[13] Radmilović V. V., Kacher J., Ivanović E. R., Minor A. M., Radmilović V. R., Multiple twinning and stacking faults in silver dendrites. Cryst. Growth Des. 16, 467–474 (2016).

[14] Zhang X., Anderoglu O., Hoagland R. G., Misra A., Nanoscale growth twins in sputtered metal films. JOM. 60, 75–78 (2008).

[15] Velasco L., Hodge A. M., The mobility of growth twins synthesized by sputtering: Tailoring the twin thickness. Acta Mater. 109, 142–150 (2016).

[16] Bufford D., Liu Y., Zhu Y., Bi Z., Jia Q. X., Wang H., Zhang X., Formation mechanisms of high-density growth twins in aluminum with high stacking-fault energy. Mater. Res. Lett. 1, 51–60 (2013).

[17] Misra A., Twinning in nanocrystalline metals. JOM. 60, 59–59 (2008).

[18] Chen M., Ma E., Hemker K. J., Sheng H., Wang Y., Cheng X., Deformation twinning in nanocrystalline aluminum. Science 300, 1275–1277 (2003). 12714676 10.1126/science.1083727

[19] Bufford D., Bi Z., Jia Q. X., Wang H., Zhang X., Nanotwins and stacking faults in high-strength epitaxial Ag/Al multilayer films. Appl. Phys. Lett. 101, 223112 (2012).

[20] Liu Q. X., El Abedin S. Z., Endres F., Electroplating of mild steel by aluminium in a first generation ionic liquid: A green alternative to commercial Al-plating in organic solvents. Surf. Coatings Technol. 201, 1352–1356 (2006).

[21] Jiang T., Chollier Brym M. J., Dubé G., Lasia A., Brisard G. M., Electrodeposition of aluminium from ionic liquids: Part I—Electrodeposition and surface morphology of aluminium from aluminium chloride (AlCl_3_)-1-ethyl-3-methylimidazolium chloride ([EMIm]Cl) ionic liquids. Surf. Coatings Technol. 201, 1–9 (2006).

[22] Agüero A., del Hoyo J. C., de Blas J. G., García M., Gutiérrez M., Madueño L., Ulargui S., Aluminum slurry coatings to replace cadmium for aeronautic applications. Surf. Coatings Technol. 213, 229–238 (2012).

[23] Fako E., Dobrota A. S., Pašti I. A., López N., Mentus S. V., Skorodumovade N. V., Lattice mismatch as the descriptor of segregation, stability and reactivity of supported thin catalyst films. Phys. Chem. Chem. Phys. 20, 1524–1530 (2018). 29260157 10.1039/c7cp07276g

[24] J. P. Hirth, J. Lothe, *Theory of Dislocations* (Krieger Publishing Company, ed. 2, 1983).

[25] Ogata S., Li J., Yip S., Ideal pure shear strength of aluminum and copper. Science 298, 807–811 (2002). 12399585 10.1126/science.1076652

[26] Qi Y., Mishra R. K., *Ab initio* study of the effect of solute atoms on the stacking fault energy in aluminum. Phys. Rev. B. 75, 224105 (2007).

[27] Poetz S., Handel P., Fauler G., Fuchsbichler B., Schmucka M., Kollera S., Evaluation of decomposition products of EMImCl·1.5AlCl_3_ during aluminium electrodeposition with different analytical methods. RSC Adv. 4, 6685–6690 (2014).

[28] Kawasaki M., Lee H.-J., Ahn B., Zhilyaev A. P., Langdon T. G., Evolution of hardness in ultrafine-grained metals processed by high-pressure torsion. J. Mater. Res. Technol. 3, 311–318 (2014).

[29] Xue S., Fan Z., Chen Y., Li J., Wang H., Zhang X., The formation mechanisms of growth twins in polycrystalline Al with high stacking fault energy. Acta Mater. 101, 62–70 (2015).

[30] Xue S., Kou W., Li Q., Fan Z., Ding J., Su R., Wang H., Zhang X., Texture-directed twin formation propensity in Al with high stacking fault energy. Acta Mater. 144, 226–234 (2018).

[31] Y. D. Gamburg, G. Zangari, *Theory and Practice of Metal Electrodeposition* (2011).

[32] Tu X., Zhang J., Zhang M., Cai Y., Lang H., Tian G., Wang Y., Electrodeposition of aluminium foils on carbon electrodes in low temperature ionic liquid. RSC Adv. 7, 14790–14796 (2017).

[33] Jovanović A., Jovanović A., Dobrota A. S., Mentus S. V., Johansson B., Skorodumova N. V., Atomic adsorption on pristine graphene along the Periodic Table of Elements – From PBE to non-local functionals. Appl. Surf. Sci. 436, 433–440 (2018).

[34] Sun F. L., Gao L.-Y., Liu Z.-Q., Zhang H., Sugahara T., Nagao S., Suganuma K., Electrodeposition and growth mechanism of preferentially orientated nanotwinned Cu on silicon wafer substrate. J. Mater. Sci. Technol. 34, 1885–1890 (2018).

[35] Hasegawa M., Mieszala M., Zhang Y., Erni R., Michler J., Philippe L., Orientation-controlled nanotwinned copper prepared by electrodeposition. Electrochim. Acta 178, 458–467 (2015).

[36] Yan T., Huang Y. C., Hou Y. C., Chang L., Epitaxial growth and microstructural evolution of nickel electrodeposited on a polycrystalline copper substrate. J. Electrochem. Soc. 165, D743–D752 (2018).

[37] Rodríguez-Clemente E., Manh T. L., Guinto-Pano C. E., Romero-Romo M., Mejía-Caballero I., Morales-Gil P., Palacios-González E., Ramírez-Silva M. T., Palomar-Pardavé M., Aluminum electrochemical nucleation and growth onto a glassy carbon electrode from a deep eutectic solvent. J. Electrochem. Soc. 166, D3035–D3041 (2018).

[38] Rozenak P., Eliezer D., Phase changes related to hydrogen-induced cracking in austenitic stainless steel. Acta Metall. 35, 2329–2340 (1987).

[39] Hermida J. D., Roviglione A., Stacking fault energy decrease in austenitic stainless steels induced by hydrogen pairs formation. Scr. Mater. 39, 1145–1149 (1998).

[40] Kireeva I. V., Chumlyakov Y. I., Tverskov A. V., Maier H., Effect of hydrogen on orientation dependence of critical shear stress and mechanism of straining in single crystals of stable stainless steel. Tech. Phys. Lett. 37, 522–525 (2011).

[41] Narita N., Altstetter C. J., Birnbaum H. K., Hydrogen-related phase transformations in austenitic stainless steels. Metall. Trans. A. 13, 1355–1365 (1982).

[42] Astafurova E. G., Zakharova G. G., Maier H. J., Hydrogen-induced twinning in 〈0 0 1〉 Hadfield steel single crystals. Scr. Mater. 63, 1189–1192 (2010).

[43] Chang J.-K., Chen S.-Y., Tsai W.-T., Deng M.-J., Sun I.-W., Electrodeposition of aluminum on magnesium alloy in aluminum chloride (AlCl_3_)–1-ethyl-3-methylimidazolium chloride (EMIC) ionic liquid and its corrosion behavior. Electrochem. Commun. 9, 1602–1606 (2007).

[44] Oliver W. C., Pharr G. M., An improved technique for determining hardness and elastic modulus using load and displacement sensing indentation experiments. J. Mater. Res. 7, 1564–1583 (1992).

[45] Giannozzi P., Baroni S., Bonini N., Calandra M., Car R., Cavazzoni C., Ceresoli D., Chiarotti G. L., Cococcioni M., Dabo I., Corso A. D., de Gironcoli S., Fabris S., Fratesi G., Gebauer R., Gerstmann U., Gougoussis C., Kokalj A., Lazzeri M., Martin-Samos L., Marzari N., Mauri F., Mazzarello R., Paolini S., Pasquarello A., Paulatto L., Sbraccia C., Scandolo S., Sclauzero G., Seitsonen A. P., Smogunov A., Umari P., Wentzcovitch R. M., QUANTUM ESPRESSO: A modular and open-source software project for quantum simulations of materials. J. Phys. Condens. Matter 21, 10.1088/0953-8984/21/39/395502 (2009). 10.1088/0953-8984/21/39/395502 21832390

[46] Perdew J. P., Burke K., Ernzerhof M., Generalized gradient approximation made simple. Phys. Rev. Lett. 77, 3865–3868 (1996). 10062328 10.1103/PhysRevLett.77.3865

[47] Monkhorst H. J., Pack J. D., Special points for Brillouin-zone integrations. Phys. Rev. B. 13, 5188–5192 (1976).

[48] Bengtsson L., Dipole correction for surface supercell calculations. Phys. Rev. B. 59, 12301–12304 (1999).

[49] El Abedin S. Z., Moustafa E. M., Hempelmann R., Natter H., Endres F., Additive free electrodeposition of nanocrystalline aluminium in a water and air stable ionic liquid. Electrochem. Commun. 7, 1111–1116 (2005).

[50] Davey W. P., Precision measurements of the lattice constants of twelve common metals. Phys. Rev. 25, 753–761 (1925).

[51] C. Kittel, *Introduction to Solid State Physics* (1996).

[52] A. Kokalj, in *Computational Materials Science* (2003), vol. 28, pp. 155–168.

[53] Momma K., Izumi F., *VESTA*: a three-dimensional visualization system for electronic and structural analysis. J. Appl. Cryst. 41, 653–658 (2008).

