# Supplementary material for: High density of genuine growth twins in electrodeposited aluminum
Source: Sci Adv. 2019 Oct 18;5(10):eaax3894. doi: 10.1126/sciadv.aax3894 (PMC6799985; doi:10.1126/sciadv.aax3894)
Supplement: http://advances.sciencemag.org/cgi/content/full/5/10/eaax3894/DC1 [file supp_5_10_eaax3894__index.html]

Science Advances | Science AdvancesAAASSearchScience AdvancesMenu

## Supplementary Materials

**This PDF file includes:**

- Fig. S1. Cyclic voltammogram recorded on Ag substrate.
- Fig. S2. Potential time curves obtained at different current densities on Ag substrates in AlCl3-EMImCl ionic liquid at 85°C.
- Fig. S3. SEM image of Al deposit formed at a current density of −10 mA cm−2.
- Fig. S4. SEM images of Al interface and screen-printed Ag substrate.
- Fig. S5. Supercell used in bulk defect calculations and different combinations of twinning.
- Fig. S6. Nanoindentation on cross sections obtained on electrodeposited Al sample and a commercial high-purity Al foil.
- Table S1. Average composition measured by EDS from the top-view TEM sample.
- Table S2. Calculated lattice constants, cohesive energies, twin formation energy, and intrinsic stacking fault energy.
- References (*49*–*52*)

Download PDF

**Files in this Data Supplement:**

- Adobe PDF - aax3894\_SM.pdf
