# Supplementary material for: High density of genuine growth twins in electrodeposited aluminum
Source: Sci Adv. 2019 Oct 18;5(10):eaax3894. doi: 10.1126/sciadv.aax3894 (PMC6799985; doi:10.1126/sciadv.aax3894)
Supplement: Download PDF [file aax3894_SM.pdf]

Science Advances
AAAS

advances.sciencemag.org/cgi/content/full/5/10/eaax3894/DC1

Supplementary Materials for

High density of genuine growth twins in electrodeposited aluminum

Lidija D. Rafailović*, Christoph Gammer, Christian Ebner, Christian Rentenberger, Aleksandar Z. Jovanović, Igor A. Pašti, Natalia V. Skorodumova, H. Peter Karnthaler

*Corresponding author. Email: lidija.rafailovic@cest.at

Published 18 October 2019, *Sci. Adv.* **5**, eaax3894 (2019)
DOI: 10.1126/sciadv.aax3894

This PDF file includes:

Fig. S1. Cyclic voltammogram recorded on Ag substrate.
Fig. S2. Potential time curves obtained at different current densities on Ag substrates in $AlCl_3$-[EMIm]Cl ionic liquid at 85°C.
Fig. S3. SEM image of Al deposit formed at a current density of -10 mA cm^{-2}.
Fig. S4. SEM images of Al interface and screen-printed Ag substrate.
Fig. S5. Supercell used in bulk defect calculations and different combinations of twinning.
Fig. S6. Nanoindentation on cross sections obtained on electrodeposited Al sample and a commercial high-purity Al foil.
Table S1. Average composition measured by EDS from the top-view TEM sample.
Table S2. Calculated lattice constants, cohesive energies, twin formation energy, and intrinsic stacking fault energy.
References (*49–52*)

Supplementary Materials

Among studies conducted on different noble (Pt, Au) or inert (W, glassy carbon) substrates it is demonstrated that the Al deposition process is controlled by nucleation kinetics (*49*). Cyclic voltammogram recorded in AlCl3-[EMIm]Cl ionic liquid on Ag substrate using three electrode set up and Al as a reference electrode is presented in fig. S1.

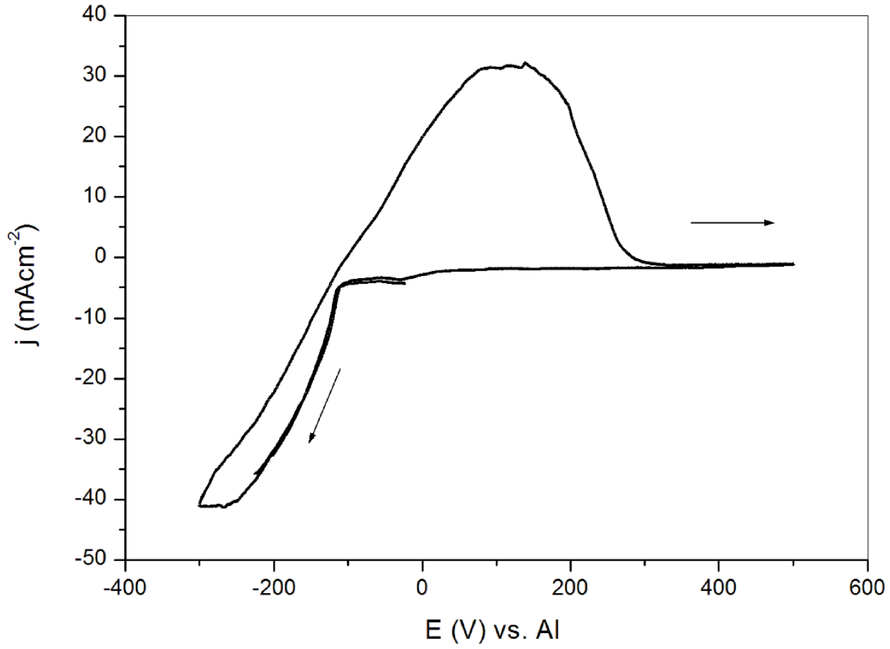

Fig. S1. Cyclic voltammogram recorded on Ag substrate. $AlCl_3$–1-ethyl-3-methylimidazoliumchloride [EMIm]Cl ionic liquid at 85 °C and scan rate: 10 mVs^{-1}.

Dense pure Al coatings are difficult to achieve since Al is a highly reactive metal with very negative standard electrode potential ($E = -1.662$ V *vs*. SHE) that cannot be deposited from aqueous electrolytes due to predominant hydrogen evolution. Therefore, Al deposition requires aprotic electrolytes and special care during processing. Chloroaluminate, $AlCl_3$-[EMIm]Cl imidazolium based ionic liquids are the most used electrolytes to achieve formation of uniform, dense microcrystalline and nanocrystalline Al coatings (21,43).

Potential-time curves obtained at different current densities on Ag substrates are shown in fig. S2.

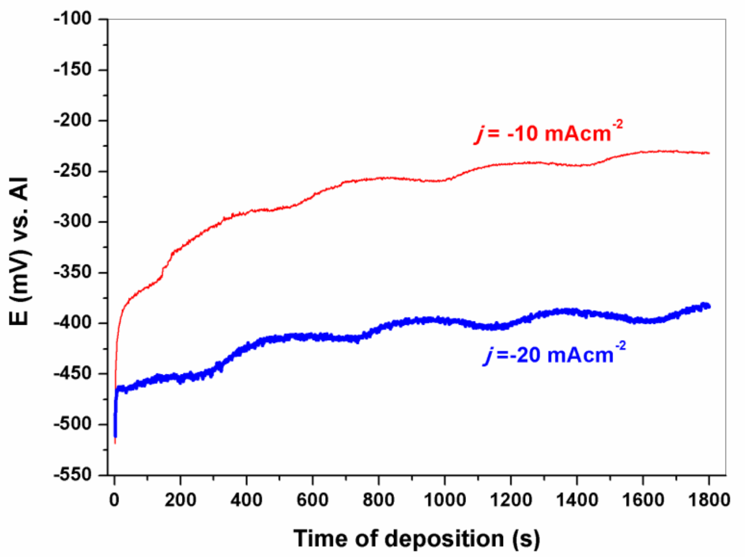

Fig. S2. Potential time curves obtained at different current densities on Ag substrates in $AlCl_3$-[EMIm]Cl ionic liquid at 85°C. Increasing current density reduces induction times and requires more negative overpotentials indicating higher nucleation rate.

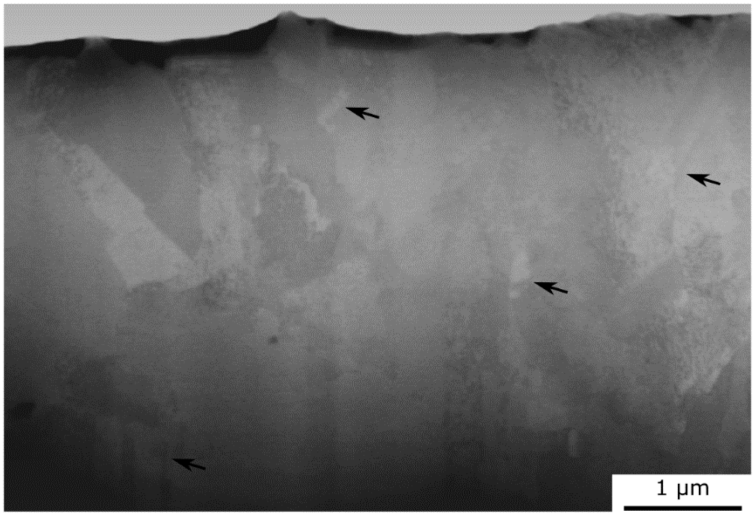

Fig. S3. SEM image of Al deposit formed at a current density of –10 mA cm^{-2}. Some twins are marked with arrows.

Although the microstructure of the electrodeposited layer is dependent on electrochemical parameters and baths used for synthesis, to date detailed structural investigations of electrodeposited Al layers are lacking (*49*). SEM image showing some twin formation during electrodeposition at lower current density of j= 10 mAcm^{-2} is presented in fig. S3. Microstructure of Ag substrate and electrodeposited Al at the interface is shown in fig. S4.

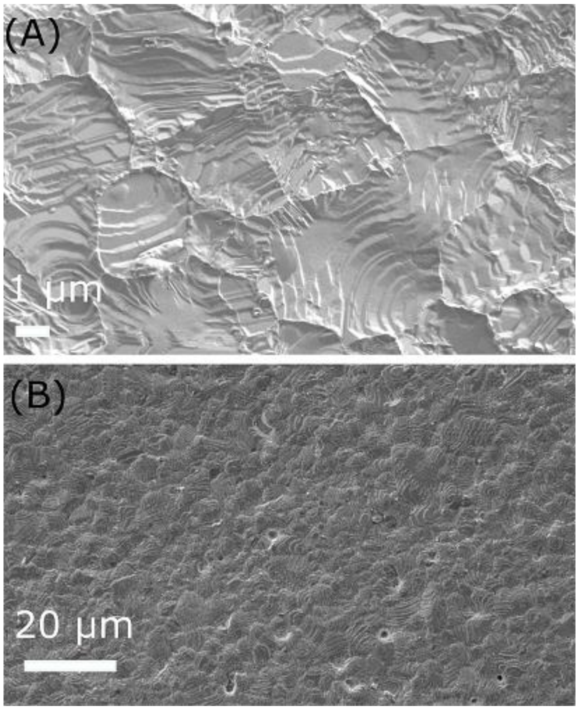

Fig. S4. SEM images of Al interface and screen-printed Ag substrate. (A) Al at the interface showing a structure copied of the fired Ag substrate; (B)Ag substrate (fired at 850°C) showing thermal etching.

To exclude segregation of any trace elements, an EDS map across a twin boundary was analysed. To reach a better statistic the map was converted to a composition profile across the twin boundary. Composition profile measured by EDS from the top-view TEM sample across a twin boundary. No compositional changes are visible at the twin boundary excluding segregation effects. The profiles indicate no variations in composition across the boundaries and all the elements are found to be homogeneously distributed across the twin boundary. It should be pointed out that all elements are present in a very low concentration apart from O due to the surface oxide layer. From these results, it can be concluded that the electrodeposited Al layer has a very high purity with only small traces of Cl.

Table S1. Average composition measured by EDS from the top-view TEM sample. Only traces of Cl can be revealed. O can be explained by oxidation, Ar through the sample preparation and the other elements can be explained by the measurement in the TEM.

**Element**	**Composition [at.%]**
Al	98 ±2
O	1.27 ±0.05
Mo	0.37 ±0.07
Cu	0.33 ±0.05
Cl	0.15 ±0.03
Ar	0.09 ±0.03
Fe	0.04 ±0.03

Comparison of the total ground state energies of the systems represented in fig. S2B revealed that the TT system is favored to the TP one, i.e. that the twin propagation from Ag to Al is energetically favorable. Partial reconstruction of TP to TT could even be observed during the structural relaxation steps.

Table S2. Calculated lattice constants, cohesive energies, twin formation energy, and intrinsic stacking fault energy.

	**a / Å**		**E_{coh} / eV per atom**		**γ_T/ mJm^{-2}**		**γ_{ISF}/ mJm^{-2}**	
	DFT	exp. (*50*)	DFT	exp. (*51*)	DFT	exp. (*24*)	DFT	exp. (*24*)
**Ag**	4.16	4.08	3.18	2.95	7.8	8	24	16
**Al**	4.04	4.05	3.57	3.39	80	75	170	166

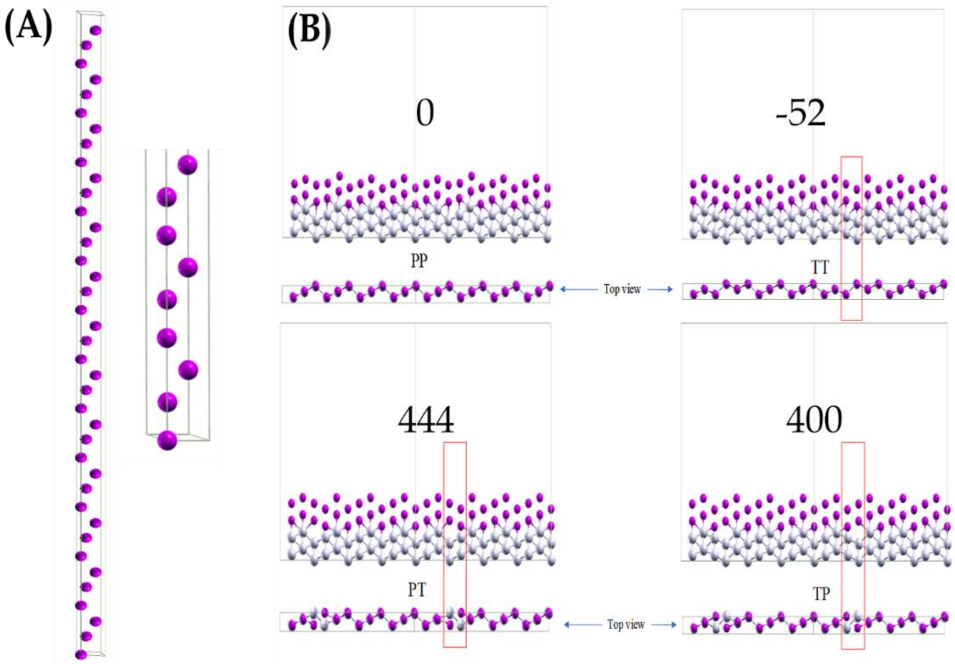

Fig. S5. Supercell used in bulk defect calculations and different combinations of twinning. (A) Hexagonal 1x1 supercell used in bulk defect calculations. (B) Four investigated combinations of twinning, shown here is the case of 6 rows of Al atoms. The thickness of the Al layer was varied from 3 to 24 atomic layers. The energy difference compared to the pristine system, in units of meV, is shown above each image. Images were generated using XCrysDen (*52*).

To investigate mechanical properties of electrodeposited Al, nanoindentation measurements were carried out. Comparison to pure Al (Good Fellow, 99.999%) was made and results are presented in fig. S6. Apart from slight deviations at low indentation depths up to 200 nm, caused by the tip rounding, no significant depth dependence for hardness or Young‘s modulus can be observed. Both samples showed some creep, resulting in a slight reduction of the measured hardness and modulus as can be seen from the last data points in fig. S3. The slightly narrower error bars in case of the commercial Al foil might be explained by the better statistics (40 measurement points compared to 25 for) for this sample.

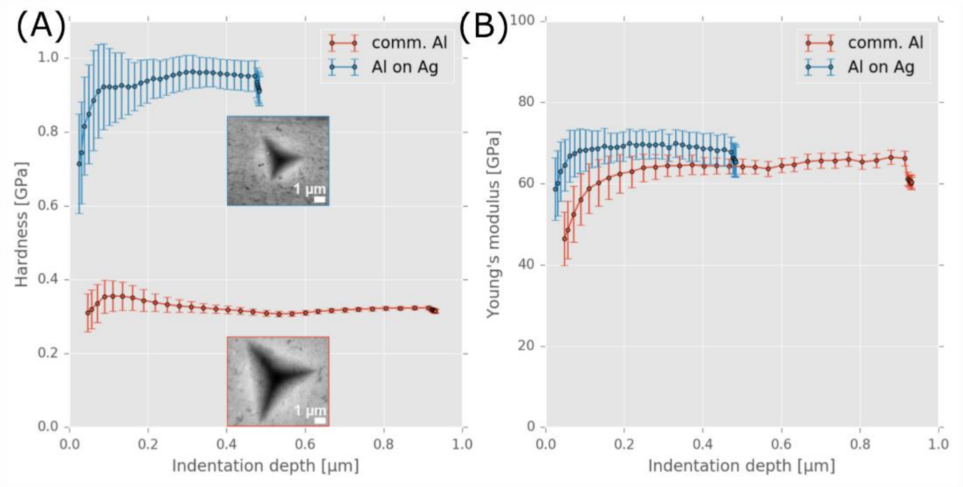

Fig. S6. Nanoindentation on cross sections obtained on electrodeposited Al sample and a commercial high-purity Al foil. (A) Nanoindentation on polished cross-sections prepared from both, the electrodeposited Al sample and a commercial high-purity Al foil. An increase in the hardness by a factor of about 3 is revealed, SEM images of an indent from both samples are shown as inserts. (B) Young's modulus is similar for both specimens.

E = 65.2±4.1 GPa for the Al layer and E = 60.3±1.7 GPa.
